# Supplementary material for: Behavioural Determinants of COVID-19-Vaccine Acceptance in Rural Areas of Six Lower- and Middle-Income Countries
Source: Vaccines (Basel). 2022 Jan 29;10(2):214. doi: 10.3390/vaccines10020214 (PMC8875839; doi:10.3390/vaccines10020214)
Supplement: Supplementary file 1 [file vaccines-10-00214-s001.zip › vaccines-1555532-supplementary.pdf]

Supplemental File: Full Results from Barrier Analysis Study

Very high levels of association (ERR ≥ 8) were found between COVID-19 vaccines acceptance and (1) perceived social norms (Bangladesh, Kenya and Tanzania), (2) perceived positive consequences (Bangladesh, DRC and Tanzania), (3) perceived susceptibility/risk of getting COVID-19 (Bangladesh and Tanzania), (4) trust in COVID-19 vaccines (DRC, Kenya and Tanzania), (5) perceived safety of COVID-19 vaccines (Kenya and Tanzania), and (6) perceived self-efficacy (DRC).

Table S1: Very high level of association between the determinants and vaccine acceptance, ERR ≥ 8.0

Question	Degree of association<br>*Est. Relative Risk > 8.0 (p<0.05)*	Behavior Determinant
If a vaccine for COVID-19 were available to ***people in your community*** *in the coming month free of charge,* ***what portion of the people you know do you think would get the vaccine****?*	Acceptors were more likely to say **most close family and friends will get the vaccine** (100% of Acceptors vs. 38% of Non-acceptors, p<0.001, **ERR>13.8)** and Non-acceptors were more likely to say they **don't know / won't say if they would** (0% Acceptors vs. 40% Non-acceptors, p<0.001, **ERR>8.3)**. (*Bangladesh)*<br>Acceptors were more likely to say that **most of their close family and friends will get a COVID-19 vaccine** than Non-acceptors (100% of Acceptors vs. 16% of Non-acceptors, p<0.001, **ERR>23.3**). *(Tanzania)*<br>Non-acceptors were more likely to say that most **community leaders** and **religious leaders would not want them to get COVID-19 vaccine** than Acceptors (0% Acceptors vs 29% Non-acceptors, p<0.001, **ERR>9.8)** *(Kenya)*<br>Acceptors were more likely to say that **most of their community leaders and religious leaders want them to get a COVID-19 vaccine** (100% of Acceptors vs. 29% of Non-acceptors, **ERR>18.3**, p<0.001). *(Tanzania)*<br>Non-acceptors were more likely to say that they would **"not [be] likely" to get a COVID-19 vaccine if a doctor or nurse recommended it** (0% of Acceptors vs. 93% of Non-acceptors, **ERR>27.1,** p=0.001). *(Tanzania)*	**Perceived Social Norms**
If a vaccine for COVID-19 were available to you in the coming month free of charge,	Non-acceptors were more likely to say that there were **"no advantages" of getting a COVID-19 vaccine** (0% of Acceptors vs. 100% of Non-acceptors, **ERR>30.3,** p=0.001). *(Tanzania)*	**Perceived Positive Consequences**

Question	Degree of association<br>*Est. Relative Risk > 8.0 (p<0.05)*	Behavior Determinant
what would be the advantages of getting that vaccine?	Acceptors were more likely to say that an advantage of getting the vaccine would be that **they would not get the COVID-19 disease** (100% of Acceptors vs. 53% of Non-acceptors, p<0.001, **ERR>9.8**). *(Bangladesh)*<br>Non-acceptors were **8.9** times more likely to say that there were "no advantages" of getting a COVID-19 vaccine (p<0.001). *(DRC)*	
*To your knowledge, **what proportion of people** in the community where you live **have had COVID-19 disease**? Would you say that very many people, some people, very few people, or no one has had COVID-19 where you live?*	Non-acceptors were more likely to say that **"very few people" in their community have had COVID-19** (0% of Acceptors vs. 87% of Non-acceptors, **ERR>24.2**, p<0.001). (*Tanzania*)<br>Surprisingly, Acceptors were more likely to say that **"no one" in their community has had COVID-19 (**100% of Acceptors vs. 13% of Non-acceptors, **ERR>24.2**, p<0.001) (*Tanzania*)	**Perceived susceptibility/ risk** (of getting COVID-19)
*How **concerned** are you about getting COVID-19? Would you say that you are not at all concerned, a little concerned, moderately concerned, or very concerned?*	Non-acceptors were likely to say that they were **"a little concerned" about getting the COVID-19 disease** (vs. other responses; 44% of Non-acceptors vs. 0% of Acceptors, ERR>9.3), and 5 times more likely to say that they were **"not concerned at all" about getting COVID-19** (p=0.002). (*Bangladesh*)	**Perceived Severity / Perceived Susceptibility**
*How much would you **trust** a new COVID-19 vaccine if it were available to you in the coming month free of charge? Would you say you would not trust it at all, trust it a little, trust it a moderate amount, or trust it a lot?*	Non-acceptors were more likely to say that they **don't trust COVID-19 vaccine "at all"** than Acceptors (0% of Acceptors vs. 51% of Non-acceptors, **ERR>12**, p<0.001**).** (*Tanzania*)<br>Non-acceptors were 17.9 times more likely to say that they would **not trust COVID-19 vaccines "at all"** (p<0.001). (*Kenya*)	**Trust** (in the vaccine)

Question	Degree of association<br><br>*Est. Relative Risk > 8.0 (p<0.05)*	Behavior Determinant
*How difficult is it for you to **get to the clinic** where vaccines are normally offered?*	Non-acceptors were **17.7** times more likely (than Acceptors) to say that it would be "**very difficult**" to get to the clinic where vaccines are normally offered (p<0.001). (*DRC*)	**Access**
*If a vaccine for COVID -19 were available to you in the coming month free of charge, what might make it **easier** for you to get that vaccine?*	Non-acceptors were more likely to say that having **"no trust in the C19 vaccines"** makes it difficult (0% of Acceptors vs. 71% of Non-acceptors, p<0.001, **ERR>18.3**). (*Tanzania*).	**Trust** (in the vaccine)
*Some people are concerned about the safety of vaccines – such as the likelihood for having a serious reaction – and some people are not. How **safe** do you think it would be for you to get a COVID-19 vaccine? Would you say it would not be safe at all, it would be mostly safe, or it would be very safe for you to get the vaccine?*	Non-acceptors were **16.8** times more likely to say that they it would **"not be safe at all" for them to get a COVID-19 vaccine** (p<0.001). (*Tanzania*).<br><br>Non-acceptors were **9.7** times more likely to say that it is **"not safe at all" to get a COVID-19 vaccine** (p<0.001). (*Kenya*)	**Safety**
*If a vaccine for COVID -19 were available to you in the coming month free of charge, what would be the **advantages** of getting that vaccine?*	Non-acceptors were **8.9** times more likely to say that there were "**no advantages**" of getting a COVID-19 vaccine (p<0.001). (DRC)	**Perceived Positive Consequences (Advantages)**
If a vaccine for COVID -19 were available to you in the coming month free of charge,	Non-acceptors were **8.9** times more likely to say that having "**no certainty about the existence of the disease because it is a plan to exterminate the African population**" would make it **difficult** to get the vaccine	**Perceived Negative Consequences**

Question	Degree of association<br>*Est. Relative Risk > 8.0 (p<0.05)*	Behavior Determinant
*what might make it **difficult** for you to get that vaccine?*	than Acceptors (p<0.001). *(This responses came up when asking about "What would make it difficult?" but is related to an expected negative consequence – extermination.) (DRC)*	
*If a vaccine for COVID -19 were available to you in the coming month free of charge, what might make it **difficult** for you to get that vaccine?*	Non-acceptors were **15.4** times more likely to say that "**I don't know (or wouldn't say)**" would ***make it easier*** to get a COVID-19 vaccine (p<0.001). (DRC)	**Perceived Self-efficacy**

Annex -2

High levels of association (ERR = 4.0-7.9) were found between COVID-19 vaccine acceptance and (1) perceived self-efficacy, (2) perceived social norms, (3) trust in COVID-19 vaccines, (4) trust in the information that community and religious leaders provide on the safety and effectiveness of COVID-19 vaccine (5) perceived access to vaccines; (6) perceived divine will, (7) perceived action efficacy (of the COVID-19 vaccines), (8) perceived positive consequences, (9) perceived negative consequences, (10) perceived severity, and (11) cultural reasons for not getting a vaccine.

Table S2: High level of association between the determinants and vaccine acceptance, ERR = 4.0 – 7.9

Question	Degree of association<br>*Est. Relative Risk =4.0 – 7.9 (p<0.05)*	Behavior Determinant
If a vaccine for COVID -19 were available to you in the coming month free of charge, what might make it ***difficult*** *for you to get that vaccine?*	Non-acceptors were **4.6** times more likely to say that the **amount of time required to get the vaccine would make it more difficult** (p<0.001).<br>Non-acceptors were **4** times more likely to say that **having a prolonged illness** would make it more difficult (p<0.001). (*Myanmar*)<br>Non-acceptors were **5.1** times more likely to say that **lack of information about the COVID-19 vaccine** would make it more difficult to take the COVID-19 vaccine (p<0.025). (*India*)<br>Non-acceptors were **4.4** times more likely to say that there was **nothing** that would make it difficult (p=0.049). (*India*)	**Perceived Self-efficacy**
If a vaccine for COVID -19 were available to ***people in your community*** *in the coming month free of charge,* ***what portion of the people you know do you think would get the vaccine****?*	Non-acceptors were **6.1** times more likely to say that **very few people they know** would get a COVID-19 vaccine than Acceptors (p=0.001). (*India*)<br>Non-acceptors were more likely to say that they would be "not likely" (vs. very likely or somewhat likely) to get the vaccine if a **doctor or nurse recommended it** (p<0.001, **ERR>4**). (*Mynamar*).	**Perceived Social Norms**
If a ***doctor or nurse recommended*** *that you get the COVID-19 vaccine, how likely would you be to get it? If a vaccine for COVID -19 were available to you in*	Non-acceptors were **4.8** times more likely to say that it was "**not likely**" that they would get a COVID-19 vaccine if a doctor or nurse recommended it (p<0.001). *(DRC)*<br>Non-acceptors were more likely to say that they **"do not know/won't say"** if the people they know would get a	

Question	Degree of association<br>*Est. Relative Risk =4.0 – 7.9 (p<0.05)*	Behavior Determinant
*the coming month free of charge, who are the people that would **approve** of your getting the vaccine?*	COVID-19 vaccine (0% of Acceptors vs. 16% of Non-acceptors, **ERR>5.3,** p=0.005) (*India*)	
*How much would you **trust** a new COVID-19 vaccine if it were available to you in the coming month free of charge?*	Non-acceptors were **7.1** times more likely to say that they **"do not trust [COVID-19 vaccines] at all"** (p<0.001). (DRC)<br>Non-acceptors were **5** times more likely to say that they **"do not trust [COVID-19 vaccines] at all"** (0.002), and **3.7** times more likely to say that they **"trust them a little"** (p<0.001), while Acceptors were **2.1** times more likely to say that they **trust the COVID-19 vaccines "a lot"** (p<0.001). (*Bangladesh*)<br>Non-acceptors were **4.9** times more likely to say that they would **trust a COVID-19 vaccine "a little"** (vs. not trust at all, trust a moderate amount, or trust a lot) (p<0.001). (*Myanmar*)	**Trust** (in COVID-19 vaccines)
Would you trust the information that religious leaders provide on the safety and effectiveness of COVID-19 vaccines?	Non-acceptors were **6.9** time more likely to say that they had a **"somewhat low level of trust"** in the information that religious leaders provided on the safety and effectiveness of COVID-19 vaccines (p<0.001). (*Tanzania*)	**Trust** (in COVID-19 vaccine information and messengers)
Do you think that there are some people or leaders who want people to get a COVID-19 vaccine as a way to either control them or harm them?	Non-acceptors were **5.1** times more likely to say that they "don't know or won't say" if there are some people or leaders who **want people to get a COVID-19 vaccine as a way to either control them or harm them.** (p=0.03). (*India*)	
How much would you trust a new COVID-19 vaccine if it were available to you in the coming month free of charge?	Non-acceptors were **7.1** times more likely to say that they do "**not trust [COVID-19 vaccines] at all**" (p<0.001). *(DRC)*	**Trust** (in COVID-19 vaccines)
If a vaccine for COVID -19 were available in the country in the coming month	Acceptors were **5.8** times more likely to say that they expect the vaccine to be **available within 30 minutes of their home** (p<0.001). (*Myanmar*)	**Access**

Question	Degree of association<br>*Est. Relative Risk =4.0 – 7.9 (p<0.05)*	Behavior Determinant
free of charge, do you think that it would be available within 30 minutes' walk from your home?<br>*If a vaccine for COVID -19 were available in the country in the coming month free of charge, how much time in minutes or hours do you think people would need to wait in queue, on average, to receive the vaccine?*	Non-acceptors were **5.2** times more likely to say that they **<u>do not</u> expect the vaccine to be available within 30 minutes** of their home (p<0.001). (*Myanmar*)<br>Non-acceptors were more likely to say that they would **expect a 31-60 min queue time** to get a COVID-19 vaccine (0% of Acceptors vs. 27% of Non-acceptors, **ERR>5.9**, p<0.001). (*Tanzania*)<br>Non-acceptors were more likely to say that they **don't know / won't say if they think that COVID-19 vaccines will be available 30 min** from their home (0% of Acceptors vs. 22% of Non-acceptors, **ERR>4.9**, p=0.001). (*Tanzania*)	
Do you think that God [or Allah or the gods] approve(s) or disapprove(s) of people getting a COVID-19 vaccine?<br>*Do you agree or disagree with the following statement? "Whether I get COVID-19 or not is purely a matter of God's will or chance. The actions I take will have little bearing on whether or not I get COVID-19."*	Non-acceptors were **4** times more likely to say that they "don't know or won't say" if **Allah approves of people getting the COVID-19 vaccines** (p<0.001). (*Bangladesh*)<br>Non-acceptors were **5.7** times more likely to say that they **believe God/ Allah/ gods "doesn't approve or disapprove"** of people getting vaccine (p=0.015). (*Kenya*)<br>Non-acceptors were **6.7** times more likely to say that they **"agree a lot"** (vs. agreeing a little or disagreeing) that **"whether I get COVID-19 or not is purely a matter of God's will or chance"** (p<0.001). (*Bangladesh*)	**Perceived Divine Will**
If you were to get the vaccine for COVID-19, how likely would it be that you would get COVID-19 disease after that?	Non-acceptors were **7.8** times more likely to say that they **did not know or would not say if they would be likely to get COVID-19 once they were vaccinated against it** (i.e. if they believed the vaccine would work, p<0.001), while Acceptors were **2.6** times more likely to say that it was **"not likely at all" that they would get COVID-19 once they were vaccinated against it** (p<0.001). (*Bangladesh*)	**Perceived Action Efficacy**
If a vaccine for COVID -19 were available to you in the coming month free of	Non-acceptors were **6.7** times more likely say that they **don't know of any advantages** of getting a COVID-19 vaccine (p=0.006). (*India*)	**Perceived Positive Consequences**

Question	Degree of association<br>*Est. Relative Risk =4.0 – 7.9 (p<0.05)*	Behavior Determinant
*charge, what would be the **advantages** of getting that vaccine?*		
*If a vaccine for COVID -19 were available to you in the coming month free of charge, what would be the **disadvantages** of getting that vaccine?*	Non-acceptors were more likely to say that **fear of is a disadvantage** of getting the COVID-19 vaccine (0% of Acceptors and 40% of Non-acceptors, p<0.001, **ERR>4.9**). (*Tanzania*)<br>Non-acceptors were more likely to say that **reduction in life expectancy is a disadvantage** (0% of Acceptors vs. 20% of Non-acceptors, p=0.001, **ERR>4.5**). (*Tanzania*)<br>Non-acceptors were **5.6** times more likely to say that "causing other dangerous diseases in the human body" would be a disadvantage of getting a COVID-19 vaccine (p=0.002). *(DRC)*	**Perceived Negative Consequences**
*How **serious** would it be if you or someone who lives in your household contracted COVID-19?*	Non-acceptors were **4.6** times more likely to say that it would be **"not serious at all" if someone in their household got COVID-19** (p=0.004) and **2.6** times more likely to say that they **"did not know or would not say" how serious it would be** (p=0.01). Meanwhile, Acceptors were **2** times more likely to say that it would be **"very serious" if someone in their household got COVID-19** (p<0.001). (*Bangladesh*)<br>Acceptors were **4.2** times more likely to say that it would be **"very serious" if someone in their household got COVID-19** (p=0.007). (*Kenya*)	**Perceived Severity**
If a vaccine for COVID -19 were available to you in the coming month free of charge, are there any cultural or religious reasons that you would not get the vaccine? (What are those reasons?)	Acceptors were more likely to say that they **did not know of any cultural or religious reasons they would not get a COVID-19 vaccine** (100% of Acceptors vs. 67% of Non-acceptors, p<0.001, **ERR>6.8**), while Non-acceptors were more likely to say that they did not know or would not say if that were so (0% of Acceptors vs. 33% of Non-acceptors, p<0.001, **ERR>6.8**). (*Bangladesh*)<br>Acceptors were more likely to say that they **had not heard of anything that would stop them or others from getting the COVID-19 vaccines** (p<0.001, ERR>**6.4**). (*Bangladesh*)	**Culture**

Question	Degree of association<br>*Est. Relative Risk =4.0 – 7.9 (p<0.05)*	Behavior Determinant
	Non-acceptors were more likely to say that they **did not know or would not say if they had heard of anything that would stop them or others from getting the COVID-19 vaccines** (0% of Acceptors vs. 24% of Non-acceptors, p<0.001, **ERR>5**). (Bangladesh)<br>Non-acceptors were **5.1** times more likely to say that they **did not know or would not say about seeing or hearing anything that would stop them or others** from seeking to get the COVID-19 vaccines (p=0.03). (*India*)<br>Non-acceptors were **4.1** more likely to say that "**These are signs of the end of time as the word of God predicts**" as a cultural or religious reason they would not get a COVID-19 vaccine (p=0.015). *(DRC)*	

Supplementary File S1

Note: This is the basic questionnaire used. Some countries chose to omit certain questions.

Group: ___Doer ___Non-Doer

**Barrier Analysis Questionnaire:**
**Intended acceptance of COVID-191 vaccines among adults living in rural communities in Kenya/ Tanzania/ Myanmar/ Bangladesh/India/DRC**

**Behaviour Statement**
Adult men and women 18 years or older of age living in rural communities have the stated intention to get a COVID-19 vaccine when one is available to them free of charge. 2

Demographic Data

Country: ____________ Interviewer's Name: __________________ Questionnaire No.: ____Date: ___/___/___

Community: _____________ Mobile Phone: ____________________

Scripted Introduction:
Hi, my name is_________. I am part of a study team looking into things related to people's interest and intention for a possible vaccination with a COVID-19 vaccine. At this point, several COVID-19 vaccines are in development and may be approved soon. The study includes a discussion of this issue and will take about 20 minutes. I would like to hear your views on this topic. You are not obliged to participate in the study, and no services will be withheld if you decide not to. Likewise, if you chose to be interviewed, you will not receive any gifts, special services, or remuneration. If there is any particular question that you do not want to answer, you can just ask me to skip the question. Everything we discuss will be held in strict confidence and will not be shared with anyone else.
Would you like to participate in the study? *[If not, thank them for their time.]*

Section A - Doer/Non-doer Screening Questions

1. What is your age?
   - ❑ a. 18 years or older (specify age in years here: ____)
   - ❑ b. Less than 18 years à *Thanks the respondent, end the interview and look for another respondent*
   - ❑ c. Don't Know / Won't say à *Thank the respondent, end the interview and look for another respondent*
2. Sex of the respondent: a.q Male b.qFemale c.qOther
3. Have you ever heard of the disease COVID-19?
   - ❑ a. Yes
   - ❑ b. No à *Thank the respondent, end the interview and look for another respondent*

---

1 It will be important to find out the local name for COVID-19 (if there is one) through discussions with local health workers and use this throughout the questionnaire.

2 Always use the vaccine according to the vaccine manufacturer's guidance.

❑ c. Don't Know / Won't say à *Thank the respondent, end interview and look for another respondent*

4. If a COVID-19 vaccine was available to you in the coming month, how likely would you be to get the vaccine, that is to go for vaccination? Would you say you are likely or unlikely to get the vaccine?
1. ***(If they say "Likely:")*** *probe further and ask:* Would you say you are Very Likely or Somewhat Likely to get the vaccine?
2. ***(If they say "Unlikely:")*** *probe further and ask:* Would you say you are Very unlikely or Somewhat Unlikely to get the vaccine?

❑ a. Very Likely / Definitely would get the vaccine

❑ b. Somewhat likely

❑ c. Somewhat unlikely

❑ d. Very unlikely / Definitely would not get the vaccine

❑ e. Don't Know

❑ f. Won't say *à Thank the person and end the interview*

[If they say "definitely", mark Very Likely. If they say, "definitely not," mark Very Unlikely.]

DOER /NON-DOER CLASSIFICATION TABLE

**DOER**<br>(**ALL** of the following)	**Non-Doer**<br>(**any ONE** of the following)	**Do Not Interview**<br>(**any ONE** of the following)
Question 1 = A		Question 1 = B or C
Question 3 = A		Question 3 = B or C
Question 4 = A or B	Question 4 = C, D or E	Question 4 = F

Group: q Doer q Non-doer

Behaviour Explanation
In the following questions, I am going to be asking you about getting a vaccine that helps prevent the COVID-19 disease. When I say this, I am talking about you going to get a COVID-19 vaccine, free of charge, from a medical worker when the government or health officials announce that an approved COVID-19 vaccine is available in your area.

Section B – Research Questions

(Perceived Self-efficacy)

1. If a vaccine for COVID -19 were available to you in the coming month free of charge, what might make it ***easier*** for you to get that vaccine?
(Write all responses below. Probe with "What else?")

2. If a vaccine for COVID -19 were available to you in the coming month free of charge, what might make it ***difficult*** for you to get that vaccine?
(Write all responses below. Probe with "What else?")

(Perceived Positive Consequences)

3. If a vaccine for COVID -19 were available to you in the coming month free of charge, what would be the ***advantages*** of getting that vaccine?
(Write all responses below. Probe with "What else?")

(Perceived Negative Consequences)

4. If a vaccine for COVID -19 were available to you in the coming month free of charge, what would be the ***disadvantages*** of getting that vaccine?
(Write all responses below. Probe with "What else?")

(Perceived Social Norms)

5. If a vaccine for COVID -19 were available to **people in your community** in the coming month free of charge, **what portion of the people you know do you think would get the vaccine**? Would you say that most people would get it, about half of people would get it, or very few people would get it?
- ❑ a. Most people would get the vaccine
- ❑ b. About half of people would get the vaccine
- ❑ c. Very few people would get the vaccine
- ❑ d. Don't Know / Won't say

[If they say everyone, choose "most." If they say "no one," mark "few people."]

6. Do you think that **most of your close family and friends would want you to get a COVID-19 vaccine** if it was available to you in the coming month free of charge? *[India chose to omit this question.]*
- ❑ a. Yes
- ❑ b. No
- ❑ c. Don't Know / Won't say

7. Do you think that **most of your community leaders and religious leaders would want you to get a COVID-19 vaccine** if it was available to you in the coming month free of charge? *[Note: This question was not assessed in the DRC.]*
- ❑ a. Yes
- ❑ b. No
- ❑ c. Don't Know / Won't say

8. If a vaccine for COVID -19 were available to you in the coming month free of charge, who are the people that ***would approve*** of your getting the vaccine?
(Write all responses below. Probe with "Who else?")

9. If a vaccine for COVID -19 were available to you in the coming month free of charge, who are the people that ***would disapprove*** of your getting the vaccine?
(Write all responses below. Probe with "Who else?")

10. If a **doctor or nurse recommended** that you get the COVID-19 vaccine, how likely would you be to get it? Would you say very likely, somewhat likely, or not likely to get it? *[Note: This question was not asked in the Kenya or India studies.]*

❑ a. Very likely
❑ b. Somewhat likely
❑ c. Not likely
❑ d. Don't Know / Won't say

(Perceived Access)

11. If a vaccine for COVID -19 were available in the country in the coming month free of charge, do you think that **it would be available within 30 minutes walk** from your home?
❑ a. Yes
❑ b. No
❑ c. Don't Know / Won't say

12. If a vaccine for COVID -19 were available in the country in the coming month free of charge, how much time in minutes or hours do you think people would need to wait in queue, on average, to receive the vaccine**?** *[Note: In the DRC, respondents were asked to rate the degree of difficulty in getting to the clinic where vaccines are normally offered instead. This question was not asked in the Kenya BA study.]*
(*Write all responses below in Hours and mins)*

___ Hours ___ mins

(Perceived Susceptibility / Perceived Risk)

13. To your knowledge, what proportion of people in the community where you live have had COVID-19 disease**?** Would you say that **very many people, some people, very few people, or no one has had COVID-19 where you live?**
❑ a. Very many people
❑ b. Some people
❑ c. Very few people
❑ d. No one
❑ e. Don't Know / Won't say

14. How **likely** do you think it is that you or someone who lives in your household will contract COVID-19 over the next three months? Would you say it's very likely, somewhat likely or not likely at all?
❑ a. Very likely
❑ b. Somewhat likely
❑ c. Not likely at all
❑ d. Don't Know / Won't say

15. How **concerned** are you about getting COVID-19? Would you say that you are not at all concerned, a little concerned, moderately concerned, or very concerned?
❑ a. Not at all concerned
❑ b. A little concerned
❑ c. Moderately concerned
❑ d. Very concerned

(Perceived Severity)

16. How **serious** would it be if you or someone who lives in your household contracted COVID-19? Would you say it would be very serious, somewhat serious, or not serious at all?

❑ a. Very serious
❑ b. Somewhat serious
❑ c. Not serious at all
❑ d. Don't Know / Won't say

(Perceived Action Efficacy / Trust [in the vaccines])

17. If you were to get the vaccine for COVID-19, how likely would it be that you would **get COVID-19 disease** after that? Very likely, somewhat likely, or not likely at all?
❑ a. Very likely
❑ b. Somewhat likely
❑ c. Not likely at all
❑ d. Don't Know / Won't say

18. How much would you **trust** a new COVID-19 vaccine if it were available to you in the coming month free of charge? Would you say you would not trust it at all, trust it a little, trust it a moderate amount, or trust it a lot? *[Note: India chose note to ask this question.]*
❑ a. Not trust it at all
❑ b. Trust it a little
❑ c. Trust it a moderate amount
❑ d. Trust it a lot
❑ e. Don't Know / Won't say

19. Some people are concerned about the safety of vaccines – such as the likelihood for having a serious reaction – and some people are not. How **safe** do you think it would be for you to get a COVID-19 vaccine? Would you say it would not be safe at all, it would be mostly safe, or it would be very safe for you to get the vaccine? *[Note: India chose to omit this question.]*
❑ a. Not safe at all
❑ b. Mostly safe
❑ c. Very safe
❑ d. Don't Know / Won't say

(Perceived Divine Will)

20. Do you think that **God (or Allah or the gods) approve(s) or disapprove(s) of** people getting a COVID-19 vaccine?
❑ a. I believe that God approves
❑ b. I believe that God does not approve
❑ c. I believe that God does not approve or disapprove
❑ d. Don't Know / Won't say

21. Do you agree or disagree with the following statement? "Whether I get COVID-19 or not is purely **a matter of God's will or chance**. The actions I take will have little bearing on whether or not I get COVID-19."
(If "Agree"): "Do you agree a little or agree a lot?"
❑ a. Agree a little
❑ b. Agree a lot
(If "Disagree"): "Do you disagree a little or disagree a lot?"
❑ c. Disagree a little
❑ d. Disagree a lot

(Culture)

22. If a vaccine for COVID -19 were available to you in the coming month free of charge, are there any **cultural or religious reasons** that you would **not** get the vaccine?
❑ a. Yes
❑ b. No à Go to question #24
❑c. Don't Know / Won't say à Go to question #24

23. What are those reasons?
(Write all responses below. Probe with "Who else?")

(Other possible correlates)

24. What level of education did you complete?
❑ a. Some primary, but did not complete primary
❑ b. Completed primary
❑ c. Some secondary, but did not complete secondary
❑ d. Completed secondary
❑ e. Some college, but did not complete college
❑ f. Completed college
❑ g. Some graduate work or completed graduate degree
❑ h. Other
❑ i. Don't know / Won't say

25. Remember, if there is any question that you do not want to answer, you can just ask me to skip the question. Would you **trust the information** that government representatives and politicians provide on the safety and effectiveness of COVID-19 vaccines?

1. ***(If "Yes", ask:)*** Would you say you have a **somewhat high level of trust or a very high level of trust** in the information that government workers or politicians provide on COVID-19 vaccines?
2. ***(If "No", ask:)*** Would you say you have a **somewhat low level of trust, or a very low level of trust** in the information that government workers or politicians provide on COVID-19 vaccines?

❑ a. Very low level of trust
❑ b. Somewhat low level of trust
❑ c. Somewhat high level of trust
❑ d. Very high level of trust
❑ e. Don't know / Won't say

26. Would you **trust the information** that religious leaders provide on the safety and effectiveness of COVID-19 vaccines?

3. ***(If "Yes", ask:)*** Would you say you have a **somewhat high level of trust or a very high level of trust** in the information that religious leaders provide on COVID-19 vaccines?
4. ***(If "No", ask:)*** Would you say you have a **somewhat low level of trust, or a very low level of trust** in that information that religious leaders provide on COVID-19 vaccines?

❑ a. Very low level of trust

❑ b. Somewhat low level of trust
❑c. Somewhat high level of trust
❑ d. Very high level of trust
❑ e. Don't know / Won't say

27. Have you seen or heard of anything that would stop you or others from seeking to get the COVID-19 vaccine (which is expected soon)?
❑ a. Yes
❑ b. No ***à End the interview and thank the respondent.***
❑ c. Don't Know / Won't say ***à End the interview and thank the respondent.***

28. What have you heard that would stop you or others from seeking to get the COVID-19 vaccine?
(List all the things below that they have heard. Say "what else?" after each response.)

[Note: In DRC, respondents were also asked if they had previously received the Ebola vaccine.]

THANK THE RESPONDENT FOR HIS /HER TIME!
